# Using Next Generation RAD Sequencing to Isolate Multispecies Microsatellites for *Pilosocereus* (Cactaceae)

**DOI:** 10.1371/journal.pone.0142602

**Published:** 2015-11-11

**Authors:** Isabel A. S. Bonatelli, Bryan C. Carstens, Evandro M. Moraes

**Affiliations:** 1 Department of Biology, Federal University of São Carlos, Sorocaba, São Paulo, Brazil; 2 Department of Evolution, Ecology, and Organismal Biology, The Ohio State University, Columbus, Ohio, United States of America; National Institute of Plant Genome Research (NIPGR), INDIA

## Abstract

Microsatellite markers (also known as SSRs, Simple Sequence Repeats) are widely used in plant science and are among the most informative molecular markers for population genetic investigations, but the development of such markers presents substantial challenges. In this report, we discuss how next generation sequencing can replace the cloning, Sanger sequencing, identification of polymorphic loci, and testing cross-amplification that were previously required to develop microsatellites. We report the development of a large set of microsatellite markers for five species of the Neotropical cactus genus *Pilosocereus* using a restriction-site-associated DNA sequencing (RAD-seq) on a Roche 454 platform. We identified an average of 165 microsatellites per individual, with the absolute numbers across individuals proportional to the sequence reads obtained per individual. Frequency distribution of the repeat units was similar in the five species, with shorter motifs such as di- and trinucleotide being the most abundant repeats. In addition, we provide 72 microsatellites that could be potentially amplified in the sampled species and 22 polymorphic microsatellites validated in two populations of the species *Pilosocereus machrisii*. Although low coverage sequencing among individuals was observed for most of the loci, which we suggest to be more related to the nature of the microsatellite markers and the possible bias inserted by the restriction enzymes than to the genome size, our work demonstrates that an NGS approach is an efficient method to isolate multispecies microsatellites even in non-model organisms.

## Introduction

Microsatellites or simple sequence repeats (SSR) have been one of the most widely used molecular markers in population and conservation genetics during the last twenty years [1,2]. Such markers are codominant, reproducible, highly polymorphic and abundant in all eukaryotes [3–5], making them one of the most valuable molecular markers at shallows levels of divergence. Despite the advances in the achievement of sequence markers and single nucleotide polymorphism (SNP) data [2,6], microsatellites still have the advantage of being multiallelic, and remains as the marker of choice for many studies such as those involving fine-scale population structure, recent demographic events, and breeding or pedigree estimation [1,4].

Next generation sequencing (NGS) has become a popular approach to developing microsatellites because it enables hundreds of loci to be identified at a reduced cost (both monetary and time) even in non-model species [2]. In particular, the cloning and the laborious sequencing required to find repeated units in a small pool of sequences can be overcome when NGS is used as a tool for marker discovery and isolation. The technology provides thousands of reads containing microsatellite loci even without a previous step for enrichment of a DNA library with targeted repeat motifs, which is usually a mandatory step in the conventional process.

The second challenge, the exhaustive laboratory work for searching polymorphic markers in the targeted and related species, could also be easily replaced by the sequencing of multiple individuals with barcode tags that allow *in silico* identification of polymorphic loci, and thus decrease the time required to screen for multi-allelic markers. Furthermore, by accessing the sequence alignment from related species and evaluating the microsatellite flanking regions, one could ensure that conservative primers are designed that would improve success in cross-species amplification tests. With the great amount of data it is also possible to select only perfect microsatellite markers (i.e. uninterrupted series of a single repeat unit), as they show greater allelic variability than compound microsatellites (i.e. uninterrupted or interrupted series of two or more repeat units), and are more appropriate to perform analyses assuming the stepwise mutation model [2,7,8].

Zalapa et al. (2012) provide a good example of the advantages provided by NGS technologies in microsatellites development. They analyzed 95 studies about development of microsatellites in plant species involving both Sanger and NGS methodologies (Illumina and 454 sequencing). A great difference was observed in the number of microsatellites isolated by Sanger sequencing and NGS technologies between the years 2010 and 2011. Comparing the results of 71 Sanger to 22 NGS studies, they found more than 40-fold increase in the ability of NGS studies to discover new microsatellites. Furthermore, 454 methodology showed a higher success of microsatellites discovered over Illumina due to the longer reads provided by the former that allow the development of longer markers as well as a more effective primer design [2].

A cost-efficient and practical strategy to develop new microsatellite markers for model and non-model organisms is the simultaneously sequencing of a reduced representation genomic library of multiple individuals or species (depending on the biological level of interest), along with a subsequent comparison of sequences from the entire sample. One useful approach to the creation of reduced representation libraries is restriction-site associated DNA sequencing (RAD-seq), which targets genomic regions adjacent to restriction sites. These fragments can then be sequenced via NGS and assembled in order to produce contigs that can be screened for genomic variation. This methodology has shown effective results for different purposes such as marker development [9], genome scans [10], population differentiation [11], and phylogeography [12,13]. A number of protocols have been published [12,14–17]; most are specific for single nucleotide polymorphism (SNP) discovery, and the best protocols include cost-effective approaches to barcoding individuals, which enables pooling of samples. As RAD-seq makes it possible to sequence the same regions across different individuals in a single sequencing run, it has inherent advantages to marker development; it efficiently replaces the cloning-sequencing-screening steps of the conventional protocol.

The *Pilosocereus* (Cactaceae) genus is subdivided in two subgenera (*Pilosocereus* and *Gounellei*) and consists of 41 recognized species. The most species-rich subgenus *Pilosocereus* is arranged into five informal taxonomic groups on the basis of morphological and geographical information [18,19]. *Pilosocereus aurisetus* group comprises eight species restricted to patches of rocky savanna vegetation (campos rupestres) in eastern South America, showing an island-like distribution. Many species of this group are under threat of extinction owing to the high endemism and destruction of their habitat by mining activities. Due to the naturally fragmented distribution and high endemism, *P*. *aurisetus* group rises as a promising system for many studies regarding the evolutionary history, population genetic and conservation among isolated populations. Previous studies revealed multiple levels of distributional fragmentation, isolation leading to allopatric differentiation, and secondary contact among divergent lineages, an evolutionary history probably related to the formation of interglacial microrefugia during the Quaternary climatic cycles [20]. However, further studies are required to better elucidate the dynamic of these events and their influence in species diversification.

Here we developed and characterized a set of microsatellite markers using a restriction-site-associated DNA sequencing (RAD-seq) protocol and 454 sequencing for multiple individuals from *Pilosocereus* genus: four species of *P*. *aurisetus* group (*P*. *aurisetus*, *P*. *machrisii*, *P*. *vilaboensis*, *P*. *jauruensis*) and the species *P*. *gounellei* (subgenus *Gounellei*). We chose the 454 platform because at the time of data collection it provided longer sequence reads than other methods, but the basic approach is applicable to any platform. We designed primers for a large number of potentially amplifiable loci for each species and performed *in vitro* polymorphism evaluation and cross-species amplification tests for 22 markers. With our data we were able to analyze the performance of RAD-seq and the use of multiple individuals and species for microsatellite discovery in a 454 platform. We also compared the nucleotide composition of the microsatellites developed by the conventional approach and NGS for *P*. *aurisetus* group and discuss the possible reasons for the lower sequencing coverage observed for microsatellites loci when compared to SNPs and anonymous nuclear markers (ANM) from the same dataset.

## Materials and Methods

### Ethics Statement

No plant samples utilized here involved endangered or protected species. Samples were either collected on public or private lands with permission of the landowners and with legal authorization of the Instituto Brasileiro do Meio Ambiente e dos Recursos Naturais Renováveis IBAMA (Sisbio # 2022310).

### Plant material and reduced genomic library preparation

We collected data from 40 individuals belonging to five species of *Pilosocereus* genus. As our primary interest was to develop microsatellite markers for species from *P*. *aurisetus* group, we used four species of this group. We sampled five individuals per population from four populations of *P*. *machrisii* (Aurora do Tocantins-TO, Alto Paraíso de Goiás-GO, Cristalina-GO and Delfinópolis-MG), two populations of *P*. *aurisetus* (Grão Mogol-MG and Mendanha-MG), and one population of *P*. *vilaboensis* (Pirenópolis-GO). For *P*. *jauruensis* we sampled four individuals from one population (Rio Verde de Mato Grosso-MS). We also included one individual of a species (*P*. *gounellei)* belonging to a different subgenus (*Gounellei*) to explore the taxonomic breadth of the application of the developed molecular markers. DNA was extracted from the root tissue ground in liquid nitrogen using a Qiagen DNeasy Plant Mini Kit (Qiagen, Hilden, Germany) according to the instructions of the manufacturer with some modifications. These modifications included two elution steps with 30 μL of the elution buffer (AE) at 65°C and the use of 13.200 rpm in all centrifugation steps. Genomic DNA was purified and concentrated using Genomic DNA Clean & Concentrator kit (Zymo Research). A reduced genomic library was prepared according to an AFLP protocol [21] modified from existing studies [22,23]. Basically we performed: (i) double digestion of DNA with the enzymes EcoRI and MseI and ligation of the adaptors, (ii) amplification of the ligation product [23], (iii) selection of amplified fragments at 500–600 bp in a 1.2% ultrapure agarose gel (Invitrogen) stained with SybrSafe (Invitrogen) and purified using a Qiagen gel extraction kit (QIAGEN), (iv) selective amplification to add the 454 adaptors and the MID barcodes (Roche) required by the Roche sequencing platform and to identify individual sequences in the posterior analyses, respectively, (v) final purification of the PCR product from the gel as described above. Library concentration and quality were evaluated using PicoGreen and Bioanalyzer (Agilent Technologies, Inc, USA). Two 454 sequencing runs were conducted by the Center for Advanced Technologies in Genomics of the University of São Paulo (São Paulo, SP, Brazil).

### NGS data processing

The sequence reads generated from 454 sequencing were converted from.fna to FASTQ files and submitted to a quality control (QC) step using scripts from the Fastx_toolkit (http://hannonlab.cshl.edu/fastx_toolkit/). We required a phred quality score of 20 and selected sequences that contained at least 90% of the individual bases above this quality score. We visualized the sequencing quality using the software FASTQC that provides metrics about Basic Statistics (number of sequences, length range of the reads and %GC), Per Base Sequence Quality (range of quality values for all bases) and Per Base Sequence Content (proportion of all bases along the sequences). After the QC step we sorted the individual libraries by barcode tags and removed the 454 adaptors and MID tags using Fastx_toolkit.

### Microsatellite selection

We built contigs and created consensus sequence for each individual using pipe1.pl and pipe2.pl scripts of QDD2.1_beta (available from http://net.imbe.fr/~emeglecz/qdd.html). QDD pipe1.pl discards sequence reads without microsatellites of the raw data, thus reducing the computational demand and the running time of the next steps. We used QDD pipe2.pl to compare sequences of each individual containing microsatellites using BLAST-2.2.25+ and the reads with very high sequence identity (>95%) were grouped into contigs and used to create a consensus file with ClustalW2. We required > 0.66 of the sequences to have the same base at a particular site before constructing the consensus sequence for a given locus. Sequence reads that did not blast to any other were classified as singletons. We identified microsatellite markers in two ways. In the first, we combined the consensus sequences of all populations from the same species to develop specific markers. In the second, the consensus sequences of our whole sample (40 individuals) were mixed together and used to create a second file of consensus sequences with QDD pipe2.pl script so the microsatellites of all individuals could be compared.

Microsatellites sequences were compared against the reference collection of GenBank, and those that matched coding regions were removed. We performed this search to avoid microsatellites linked to regions under selection once our main goal was to identify neutral markers for future applications. The software MSATCOMMANDER version 1.0.8-beta [24] was used to identify perfect dinucleotides and trinucleotides motifs with at least 6 repeats in the consensus sequences. We also looked for tetra-, penta- and hexanucleotide motifs with at least three repeats. The microsatellites found in the consensus sequences were checked in the individual sequences to certify that all the individuals have the motifs identified and to discard putative paralogous loci. We consider a putative paralogous locus when we found more than two alleles occurring in the same individual, since our species are diploid [25].

Specific primers for *in vitro* evaluation were developed for 64 putative microsatellite markers that were found in more than one individual. We also provide primers for an additional set of 72 potentially amplifiable loci in the supporting information (S1 Table). Primers were designed using PRIMER3 v4.0.0 (http://bioinfo.ut.ee/primer3/) with microsatellite repeats as target regions, PCR products sizes from 100 to 250 bp, minimum, optimum, and maximum primer sizes set as 18, 20, and 25; optimum, and maximum T_m_ set at 58.0 C, 60.0 C, and 63.0 C; maximum difference in T_m_ of 2.0 C; minimum, optimum, and maximum GC content set at 20%, 50%, and 70%; and designated a GC clamp to the 3’ end, when possible. We also check for self-complementarity and hairpins for all primers in Oligocalc (http://www.basic.northwestern.edu/biotools/oligocalc.html).

### Microsatellite validation

Amplification reactions were performed in 12 μl containing 5–40 ng DNA, 1X PCR buffer (100mM Tris-HCl, pH 8.3, 500mM KCl), 1.0–1.5 mM MgCl_2_, 0.1μM each primer, 0.2 mM dNTPs, and 0.6U of JumpStart Taq DNA polymerase (Sigma-Aldrich). Primer pairs were initially evaluated in a touchdown program [26]. For some loci the annealing and elongation steps were reduced to 40 sec and 1 min, respectively (Table 1). When necessary, a specific annealing temperature was set by performing a gradient temperature program as follow: initial denaturing step at 94°C for 2 min, 30 cycles at 94°C for 1 min, 55–61°C for 1 min (optimized annealing temperatures in Table 1), and 72°C for 2 min, with a final elongation step at 72°C for 5 min. Amplification products were visualized on 3% agarose and 6% polyacrylamide gels.

**Table 1:** Characteristics of 22 PCR validated microsatellite markers developed for *Pilosocereus*.

Locus	Repeat Motif	Primer sequence (5'-3')	*Ta* (°C)	Size (bp)
*PaSSR109*	(ATC)_5–7_	F: CCCATGATGACGTTGGTGATAA	TD	94–115
		R: TCAGAATAATTACAACAGAGG		
*PaSSR139*	(TGT)_8_	F: ATGAAAATGTTGCCCTCCAC	TDred	142–156
		R: TCGCCACCTAGTGGAGAATC		
*PaSSR140*	(GGT)_6_	F: GACACCTCCTCAGGGACAAC	TDred	131–140
		R: GTTGAGGCCATCAAGGAGAC		
*PaSSR179*	(AGA)_7–9_	F: AGAGTACTCCTCCGAGCTGATTC	TDred	152–179
		R: ACTCAACGCGTCTTCTATCAAAC		
*PaSSR192*	(AAT)_6_	F: CAATCCCTCTTGAATCTCACGTG	TDred	180–195
		R: AGCGGGTTCAAGATCGAATC		
*PaSSR195*	(CAA)_7–13_	F: CCTAGACTTGGACTAAGAAGCG	61	192–216
		R: CTACCTTCAAAGCCTCTAAATCTG		
*PaSSR197*	(GAA)_6–8_	F: GTAACTTCATGACAGGCATAAG	57	176–179
		R: CGAGTTGTGATTGAGCATGG		
*PaSSR224*	(GAA)_12–15_	F: TAGGATTTATAACCAGCCCTGTC	61	179–221
		R: TGAATTACTGTTCCTATCTGTCTG		
*PaSSR150*	(GA)_9–10_	F: TTCCGGATTAGATTCGAACG	62	138–176
		R: TTCTCAAGGCCGGAAAATAC		
*PaSSR153*	(CT)_10_	F: ACTGGGAGTGGCTGAAATTTGTTAGT	61	153–181
		R: GGGAGGCTCTATTGCACCATCCATGT		
*PaSSR156*	(GA)_6–8_	F: GGATACTCTGTCGCCTGTTTCG	TDred	148–154
		R: TGGCAGYCAGTAGCAGTCAC		
*PaSSR162*	(AT)_6–7_	F: TAGAGGAGCAAAGATCAGAAAGG	TDred	158–174
		R: CCATGAGAGCCAACTCTAGAAAG		
*PaSSR164*	(TG)_6–7_	F: AATTCGGTTGGGAGCTTTTAG	61	160–183
		R: CAAGATATTCGAGCAGCAAGTAA		
*PaSSR176*	(TG)_8–15_ (CG)_4–7_	F: GTAAGCAGCCTAACCTGAATTTG	50	138–176
		R: ATGTAGAAGCATCATCATGCC		
*PaSSR181*	(CT)_10–11_	F: GGCAAGTCCACCGCCAAGAAGA	TD	167–185
		R: ACAGAACAGAAAATCGACGATGG		
*PaSSR184*	(AG)_12–13_	F: GTGCTGTTGTTCCTCACGAC	61	172–188
		R: GTTGCCCGCTGGCTCTCTC		
*PaSSR187*	(TG)_10–12_	F: GCCGGAACTTTGAAGCTTAGTG	TDred	185–213
		R: CCCATACCCATAGTCATCGGATG		
*PaSSR189*	(GA)_10–11_	F: GGTTTCAGCTCAATGGACAAC	TDred	179–189
		R: TTGCTATAACAACCTTTCCTGC		
*PaSSR190*	(CT)_8–10_	F: CCCTGAACCTGCTTCTATGC	TDred	186–200
		R: ACCGTCACACCTAACCAGC		
*PaSSR191*	(CT)_6–8_ (GT)_2_	F: GCATTTTCTGTAAGACACCTTGC	TD	177–191
		R: TTCACTTCTCCAAGACTGGATTC		
*PaSSR193*	(GA)_7–9_	F: CTCCTAGCAAGGTACGGGTAAC	TD	177–193
		R: ATGTTTGAGCTTCGATCCACAAG		
*PaSSR205*	(GA)_9–18_	F: GCAGACTTACAAAAGGGAGACTG	61	203–221
		R: CTACTGATGAATCCAGGTGAGAG		

TD: *Ta* ranging from 65–55°C in the *touchdown* PCR protocol. TDred: *touchdown* protocol with 40 sec annealing and 1min elongation.

To evaluate the polymorphism in the selected loci we used two populations of *P*. *machrisii*: Cristalina-GO (CRI) and Alto Paraíso de Goiás-GO (APA1). Individuals were genotyped on a Fragment Analyzer Automated CE System (Advanced Analytical Technologies, Ames, IA, USA) using the 35–500 base pair dsDNA Reagent Kit (Advanced Analytical Technologies, Ames, IA, USA) and the allele scoring was carried out using the software PROSize v. 2.0. Microsatellite loci were assessed for null alleles, stutter, and large allele drop-out using Microchecker v2.2.1 [27].

Linkage disequilibrium among all pairs of loci within each population and departures from Hardy–Weinberg equilibrium (HWE) were assessed by the Markov-chain approximation of the Fisher’s exact test implemented in GENEPOP v. 4.2 [28,29]. The diversity indices as number of different alleles (*N*_a_), effective number of alleles (*N*_e_), and observed (*H*_O_) and expected (*H*_E_) heterozygosities were computed using GenAlEx v. 6.501 [30] and the fixation index was estimated in FSTAT [31]. All selected loci were PCR tested in two individuals from the species *P*. *vilaboensis*, *P*. *aurisetus* and *P*. *parvus*, which will be used in further studies.

In order to identify the level of relatedness among individuals in the sample we computed the kinship coefficients for all pairs of individuals using the statistic of Loiselle *et al*. (1995) in the SPAGEDI program [32] The kinship ***θ*** *xy* parameter of Loiselle *et al*. (1995) estimates the relative similarity between individuals x and y relative to the mean genetic similarity between random individuals in the sample [33]. We chose the Loiselle *et al*. (1995) estimator because it has a higher accuracy than other kinship coefficients and makes no assumption regarding Wright’s inbreeding coefficient.

## Results

### 454 Pyrosequencing dataset

The two sequencing runs of the 40 libraries generated 2,282,266 sequence reads with an average size of 483 bp. The number of reads for each run was very similar, 1,141,399 and 1,140,867 reads per run. The QC step recovered approximately 82% of the initial reads with an average length of 450 bp (Table 2). After barcode trimming an average of 37,777 reads was obtained per individual. We recovered 703,506 sequence reads for *P*. *machrisii*; 408,408 for *P*. *aurisetus*; 185,593 for *P*. *vilaboensis*; 166,096 for *P*. *jauruensis*, and 47,477 for *P*. *gounellei*. Approximately 20% of the sequences were unmatched by any of the barcodes.

**Table 2:** Statistics of microsatellite markers developed from RAD-seq data.

Total no. of raw reads	2,282,266
Total no. of filtered reads	1,877,074 (82.25%)
Sequences longer than or equal to 80 bp after adapter clipping	1,440,911
No. of reads containing microsatellites	54,420
No. of consensus sequences among individuals containing microsatellites	623
Primers pairs screened	64
Microsatellites selected for characterization	22

### Microsatellite repeat content and loci selection

We identified an average of 165 consensus sequences per individual containing microsatellites, resulting in a combined consensus file of 6,609 sequences for all species. The total number of consensus sequences with microsatellite repeats per species was 383 for *P*. *machrisii*, 230 for *P*. *aurisetus*, 148 for *P*. *jauruensis*, 127 for *P*. *vilaboensis* and 135 for *P*. *gounellei*. The frequency distribution of the repeated units was very similar among species; dinucleotide motifs were the most abundant (ranging from 62.68% to 66.89%), followed by trinucleotides (21.83–29.76%). Longer repeats like tetra- (2.03–9.15%), penta- (0.6–2.82%) and hexanucleotide (1.27–3.52%) motifs were the least frequent microsatellites in our dataset.

**Fig 1:**
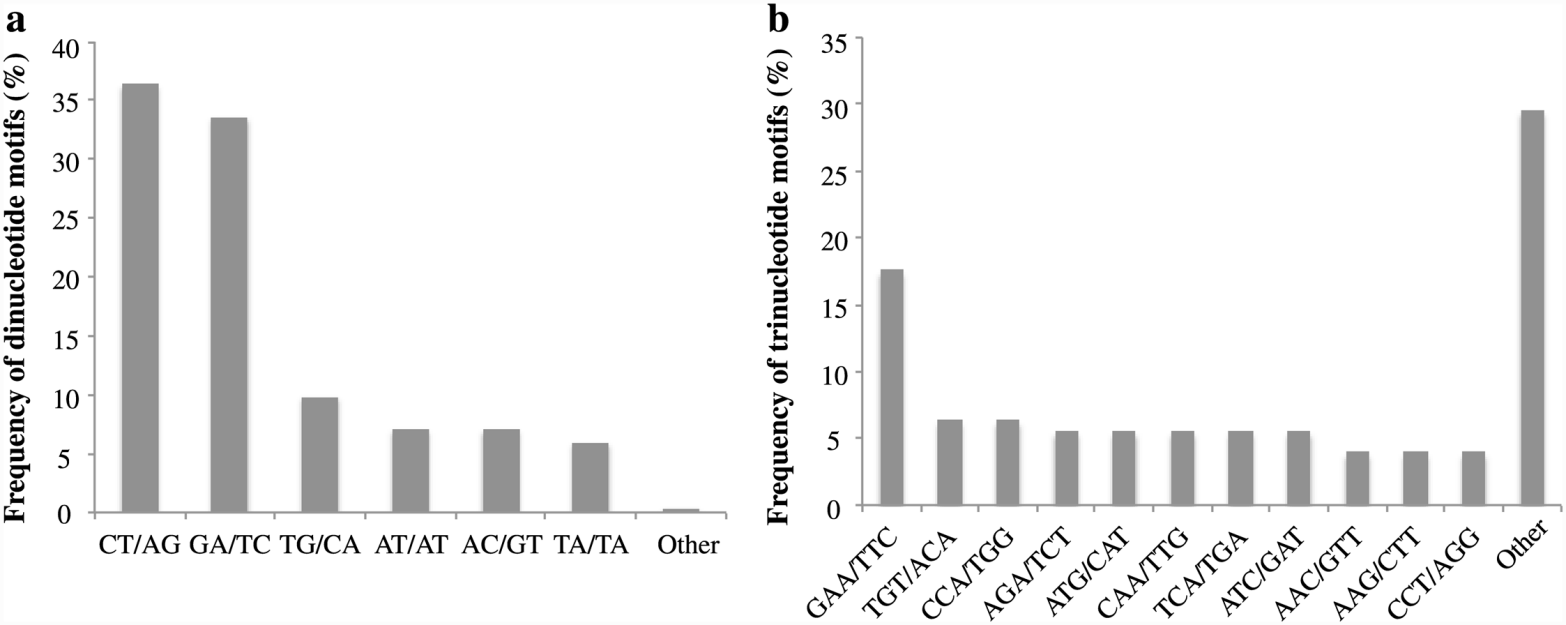
Frequency of consensus sequence containing the most abundant dinucleotide (a) and trinucleotide (b) motifs found in five species of *Pilosocereus* genus.

Analyzing all species together we obtained 623 consensus sequences containing microsatellites in more than one individual (Table 2). The dominant dinucleotide repeat motif was CT/AG (36.30%) and the least abundant were GC/GC (0.19%). We observed a great variety of trinucleotides; GAA/TTC was the most frequent repeat (17.6%), more than two-fold over the second more frequent motif TGT/ACA. The majority of the trinucleotides were less than 5% frequent in the dataset (Fig 1). We found only 10 tetranucleotide (ATTC, TTAT, CAAG, TGTT, ATAA, TTTG, ATCA, ATGT, GTAT and CAGA), 7 pentanucleotides (ATTTT, TTCTT, ACCCG, TGGTT, CCGGT and TTTGT), and 8 hexanucleotides motifs (GCAGGT, AGAGGA, GCCTCA, TGACCC, TGTATG, TCTTTT, CAAGCT and AAGGAG), the great majority occurring only once in our data.

We selected the most abundant and variable microsatellites in our *in silico* screening for polymorphic loci, which were di- and trinucleotide motifs. We found 215 perfect dinucleotides and 42 perfect trinucleotides in our search using the software MSATCOMMANDER. However, after a manual checking, we observed that some markers were, in fact, interrupted or compound in some way or did not contain appropriate flanking regions to design primers, which led us to discard these regions. After this step, we selected 64 loci for PCR validation. Only in a few cases we observed the same microsatellite locus in more than one individual of the same population.

### Microsatellites characterization

Of the 64 loci tested, 28 were effectively amplified across the four analyzed species of the *P*. *aurisetus* group. Among these, 22 loci exhibited an ideal amplification pattern (i.e., without smear and stutter bands) and were selected for the evaluation of polymorphism in two populations of *P*. *machrisii*. These loci were genotyped for 44 individuals, and produced a total of 142 alleles. Only the locus *PaSSR189* was monomorphic in population CRI, the remaining loci were polymorphic in both populations of *P*. *machrisii*. The number of alleles per locus ranged from 2 (*PaSSR197*) to 10 (*PaSSR150*) with an average of de 4.6 (*SD* ± 0.3) alleles per locus (Table 3). The largest number of alleles (> 8 alleles) was found in the loci *PaSSR150*, *PaSSR153*, *PaSSR176*, *PaSSR179* and *PaSSR224* and the lowest number (< 4 alleles) in *PaSSR139* and *PaSSR197*. The average of the effective number of alleles over loci was 2.3 (*SD* ± 0.2) in CRI and 3.2 (*SD* ± 0.3) in APA1, ranging from 1.0 (*PaSSR189*) to 5.0 (*PaSSR190*) in CRI and 1.2 (*PaSSR197*) to 6.0 (*PaSSR176*) in APA1. The average *H*_*O*_ across loci ranged from 0 to 1 (mean = 0.549, *SD* ± 0.068) within CRI and 0.100 to 1 (mean = 0.595, *SD* ± 0.052) within APA1 whereas *H*_*E*_ ranged from 0 to 0.799 (mean = 0.500, *SD* ± 0.041) within CRI and 0.180 to 0.832 (mean = 0.618, *SD* ± 0.038) within APA1. Departures from HWE were found in seven loci within population CRI (*PaSSR109*, *PaSSR187*, *PaSSR190*, *PaSSR191*, *PaSSR192*, *PaSSR193*, *PaSSR205*) and six within population APA1 (*PaSSR156*, *PaSSR181*, *PaSSR189*, *PaSSR190*, *PaSSR192*, *PaSSR193*) after Bonferroni correction (α = 0.05). Null alleles were identified in six loci for CRI and four loci for APA1, among which only two were common between populations (*PaSSR181* and *PaSSR205*). For most loci the detected presence of null alleles was a clear effect of Hardy-Weinberg disequilibrium within populations. Linkage disequilibrium was not significant among loci, so we assumed all loci to be segregating independently. The 22 primer pairs were tested in other three species of the *P*. *aurisetus* group (*P*. *aurisetus*, *P*. *vilaboensis and P*. *parvus*) using two individuals from each one. All loci successfully amplified in these species with the same PCR protocol used for *P*. *machrisii*.

**Table 3:** Genetic diversity estimated on 44 individuals of *P*. *machrisii* sampled from two populations (CRI and APA1). Presence of null alleles (Null), number of alleles (*A*), number of effective alleles (*A*_E_), and observed (*H*_O_), expected (*H*_E_) heterozygosity, and fixation index (F) are shown.

Locus	CRI							APA1						
	N	Null	*A*	*A*_E_	*H*_*O*_	*H*_*E*_	F	N	Null	*A*	*A*_E_	*H*_*O*_	*H*_*E*_	F
*PaSSR109*	21	-	3	2.2	0.870	0.547*	-0.574	14	-	6	5.1	0.895	0.803	-0.087
*PaSSR139*	22	-	2	1.2	0.087	0.159	0.470	20	-	3	1.3	0.200	0.224	0.131
*PaSSR140*	21	+	2	1.6	0.125	0.395	0.695	19	-	4	2.5	0.800	0.599	-0.313
*PaSSR150*	20	+	7	3.2	0.542	0.692	0.237	15	-	10	4.0	0.650	0.751	0.160
*PaSSR153*	22	-	5	2.7	0.583	0.626	0.089	18	-	7	5.0	0.650	0.8	0.212
*PaSSR156*	22	+	4	3.9	0.458	0.744	0.402	20	-	3	2.3	0.950	0.574*	-0.641
*PaSSR162*	23	-	2	1.5	0.292	0.353	0.195	18	-	3	1.7	0.450	0.421	-0.043
*PaSSR164*	23	-	5	2.5	0.792	0.597	-0.306	20	-	4	2.0	0.350	0.499	0.321
*PaSSR176*	23	-	2	1.4	0.375	0.305	-0.211	18	-	9	6.0	0.737	0.832	0.141
*PaSSR179*	24	-	4	2.1	0.708	0.518	-0.348	20	-	9	3.1	0.550	0.678	0.213
*PaSSR181*	23	+	3	1.4	0.125	0.284	0.574	19	+	8	4.8	0.500	0.790*	0.389
*PaSSR184*	22	+	4	2.0	0.292	0.496	0.429	20	-	3	2.1	0.400	0.521	0.257
*PaSSR187*	23	-	6	2.5	1.000	0.595*	-0.668	17	-	4	1.9	0.600	0.468	-0.260
*PaSSR189*	22	-	1	1.0	0.000	0.000	-	19	+	5	3.2	0.250	0.686*	0.651
*PaSSR190*	23	-	6	5.0	0.958	0.799*	-0.178	19	-	4	2.7	0.737	0.632*	-0.140
*PaSSR191*	24	-	3	2.1	1.000	0.520*	-0.920	20	-	4	2.7	0.850	0.629	-0.329
*PaSSR192*	22	-	2	2.0	1.000	0.500*	-1.000	20	-	4	2.2	1.000	0.548*	-0.818
*PaSSR193*	20	-	2	1.9	0.833	0.486*	-0.704	17	-	7	4.4	0.800	0.773*	-0.010
*PaSSR195*	20	-	5	2.1	0.583	0.524	-0.092	13	-	5	3.6	0.650	0.719	0.121
*PaSSR197*	22	-	2	2	0.458	0.492	0.090	19	-	2	1.2	0.100	0.180	0.465
*PaSSR205*	24	+	5	2.4	0.375	0.589*	0.382	18	+	7	4.0	0.526	0.749	0.322
*PaSSR224*	21	-	9	4.3	0.625	0.768	0.207	18	+	7	3.7	0.450	0.729	0.404
Overall	-	-	3.8	2.3	0.549	0.500	-0.078	-	-	5.4	3.2	0.595	0.618	0.063

- and + indicate absence and presence of null alleles, respectively;

* indicates significant departure from Hardy–Weinberg equilibrium after Bonferroni correction at α = 0.05

### Genetic Relatedness

According to the kinship coefficient there was no relatedness among individuals belonging to different populations. On the other hand, we detected high levels of relatedness between individuals within populations suggesting that mating among relatives may occur in these populations. The average coancestry coefficient within populations was 0.105 (*SD* ± 0.07) in CRI and 0.143 (*SD* ± 0.083) in APA1. We observed high values of coancestry: 72% of the values ranged between 0.062 to 0.356 within population CRI and 82% ranged between 0.062 to 0.449 in population APA1 (S3 Table). Such values are expected for cousins (***θ*** *xy* = 0.0625) and self-sibs (***θ*** *xy* = 0.5). However the presence of inbreeding was not recovered by the fixation index (F) within populations (Table 3). This result might be related to the excess of heterozygous in the sample that can mask the kinship among individuals.

## Discussion

### Conventional *vs* NGS microsatellites development

We observed a marked difference in the microsatellites obtained by NGS when compared with those developed by conventional methods using an enriched DNA library and Sanger sequencing for species of *P*. *aurisetus* group [34]. Although many clones were sequenced in this conventional approach (94 inserts), only three sequences showed perfect motifs. This brings a limitation to the application of these conventional developed markers in analyses adopting the step-wise mutation model, as compound and interrupted microsatellites may show more complex molecular evolution. Furthermore, non-perfect loci usually show less allelic diversity than perfect loci [7,8].

Another important advantage of NGS over the conventional development is the simultaneous isolation of microsatellites from multiple species. One great value of using multiple species for screening molecular markers is the possibility to evaluate length polymorphisms in the flanking regions and avoid genotyping errors by choosing the best position of the primers. Conventional microsatellite development methods typically generate sequence from a single specimen and do not consider the presence of repetitive elements and insertion-deletion mutation flanking the candidate locus, which can affect genotyping success and accuracy. In many cases, we observed deletions, which were present in the flanking regions of the microsatellite loci, and as such could result in misleading genotypes. Similarly, repeated regions like nanosatellites (short repetitions) or homopolymers could also promoted insertion-deletion mutations not related to the target locus leading to genotyping errors. Furthermore, by designing primers consistent for multiple species researchers could skip cross-transferability tests, which is mandatory for many studies.

### Choice of the restriction enzymes

Traditional RAD-Seq methodology uses one restriction enzyme and random shearing to generate genomic fragments. However this strategy came with high levels of DNA loss and little control over the sequenced fragments, mainly for organisms without a reference genome [35]. The use of two enzymes in the methodology (double digest RAD-seq) has overcome these shortcomings and increased the sequencing of the same genomic regions across individuals. Nowadays there are many options of restriction enzymes suitable for different NGS platforms.

The choice of enzyme has a considerable impact on number of fragments obtained [21,35]. Some facilities provide a previous *in silico* analysis to predict the coverage of the sequencing based on the genomic content of the organism. To select the best enzymes the researcher should have in mind the number of markers required and some information about the genetic diversity of the target organism. A shallow estimation of the number of restriction sites can be obtained based on the genome size and GC content [35]. This brings a bigger challenge for studies with non-model organisms such as the cactus species presented here. A good start is to use two types of enzymes, one with a frenquent cut and one with an infrequent cut to try to recover the same regions for different species. In general, species with low genetic diversity will require an enzyme with a higher cutting frequency to produce sufficient polymorphic markers. On the other hand, species with high genetic diversity are prone to show mutations in the restriction sites, which can cause sequencing coverage loss [35]. Futhermore, the choice of a restriction enzyme that produces more fragments will demand more efforts to perform the analysis. The cost benefit should be evaluated, in many cases, the researchers’ budget is important in selecting the appropriate enzyme.

### Choice of the NGS platform

Until recently, the 454 Roche platform was the most popular NGS technology for isolating microsatellites due to the longer reads produced (350–600 bp). However the total number of reads produced by the Illumina technologies was always higher and cheaper than the Roche platform. Despite the shorter reads, successful results for microsatellite isolation have been obtained on the Illumina platform, particularly from transcriptome sequencing [5,36,37]. Of the Illumina platforms, the MiSeq desktop sequencer has increased the length of the reads (2 x 300 paired-end reads) and offers the ability to produce small (and thus inexpensive) runs that lead us to suspect that it represents the best available choice for this approach.

### Bioinformatic analyses

A variety of software is available to identify microsatellites in NGS datasets (MISA, palfinder, MSATCOMMANDER, QDD), and all of these can effectively process data from 454 and Illumina platforms. QDD is an open acess program, and among the most user-friendly and versatile of the software currently available. Although it was not developed specially to work with sequences from different individuals and species, it was easily modified to do so in our analysis. When the program generates the consensus sequences (putative loci) it is possible to visualize all the sequence reads (individuals) that show the loci, which allows a clear identification of polymorphic loci. The only drawback of the software, which is common to the others available, is that it lacks the ability to select only perfect microsatellites. To this end, we used MSATCOMMANDER in which it is possible to search for only perfect microsatellites, but we still observed the occurrence of compound and interrupted repeats. This observation suggests that, even selecting the option of perfect loci, it is necessary to manually check the microsatellites to certify that we are dealing with perfect motifs.

### Sequencing coverage

We used a RAD-seq protocol with a size selection step in order to improve the coverage of the loci. Although we observed a satisfactory coverage per locus within individuals, the same was not true for the coverage across individuals. Most loci were present in a few individuals and no locus was common for all species in the dataset. If the low coverage was due to a large genome size several possible approaches can be used to further reduce the representation of the genome [12]. For example, a smaller size fragment can be excised from the agarose gel, additional selective bases can be used in the selective PCR, or use less common restriction enzyme can be used in the initial digestion. However, in our case the low coverage effect was less pronounced for SNPs and ANMs developed from the same dataset analyzed here in which a mean of 19.24 (*SD* ± 5.57) individuals containing the same locus was observed (Manolo F. Perez, pers. comm). One possible reason for this low-coverage drawback could be the mistakes inserted in sequences due to the repeated units, which may have resulted in low quality sequences excluded in the quality control step of our analysis. While type of error is well known for homopolymers [38], it appears that we observed the same effect on dinucleotide repeats. Some of the sequence reads ended within repeated regions, suggesting that the sequencing process may have been interrupted in this point.

One could also argue that the low coverage across individuals could be associated with the RAD-seq protocols. When the pool of individuals used in the RAD-seq protocol show high levels of polymorphism, it is possible that some alleles drop out due to mutations at the restriction sites. For example, variation in the restriction site among individuals could result in the presence of longer alleles for some individuals, which were excluded in the size selection step [35]. Taking into account that microsatellites are usually present in genome regions free of selection pressure, they are more susceptible to have variation in restriction sites being difficult to recover the same locus for all individuals sampled. However, missing data for some species could be easily replaced using conventional Sanger sequencing, for example. Although we do not yet know the genome size of any species of *P*. *aurisetus* group, other cactus species with the same chromosome number (2n = 22) have been reported to have small or intermediate nuclear genomes ranging from 1,565 Mbp in *Leptocereus quadricostatus* to 13,594 Mbp in *Mammillaria rhodantha* (http://data.kew.org/cvalues/). The small genome size of these cactus species led us suggest that the low coverage observed in our data might be more related to the nature of the microsatellite markers and the possible bias inserted by the restriction enzymes than to the genome size.

### Microsatellites content and polymorphism

A common characteristic of the microsatellites in our analyses was the higher variability and frequency of dinucleotides when compared to trinucleotides and longer motifs. Although trinucleotides could be a better choice because they are less prone to polymerase mistakes as slipped-strand mispairing (stuttering) and consequently genotyping errors [7,8], our *in silico* search recovered only a few polymorphic trinucleotides. This reason made us selected the most polymorphic markers, dinucleotide in most cases that will contain the genotypic variation necessary for further analysis.

### Microsatellite *vs* SNP markers

Despite the increasing popularity of SNPs for phylogeographic and landscape genetic studies [39], microsatellites remain one of the most informative and versatile markers available for genetic investigations into landscape-level questions. For most investigations, microsatellites are the most appropriate marker and traditional barriers to identifying such markers can be overcome by new technologies of sequencing. Additionally, the abundance of microsatellites in eukaryotic genomes allows these markers to be developed from other sources of data (e.g., genomes, transcriptomes).

## Conclusions

Here we provide an example of the advantages and disadvantages of using a RAD-seq protocol for the identification of microsatellite loci in a group of cactus species. Despite the difficulties that the repeated units can bring to the analysis of multiple species data, the development of microsatellites from RAD-seq data was effective for isolating new markers from non-model species, even without an enrichment library step. Futhermore, the same RAD-seq data also can serve as a source for additional molecular markers (e.g., SNPs and ANMs), as well as serving as a target for genome scans for loci under selection. In the future, the use of multiple individuals and strategies as genotyping by sequencing (GBS) will also improve microsatellite applications and enable an even more cost-effective procedure for population genetic studies.

## Supporting Information

S1 Table Potentially amplifiable microsatellites for five species of *Pilosocereus* genus. (XLSX)

S2 Table Coverage of RAD-seq and microsatellites discovery per sample. (DOCX)

S3 Table Pairwise kinship coefficiente of Loiselle et al. 1995 within populations. (XLSX)
