# Supplementary material for: Using Next Generation RAD Sequencing to Isolate Multispecies Microsatellites for Pilosocereus (Cactaceae)
Source: PLoS One. 2015 Nov 11;10(11):e0142602. doi: 10.1371/journal.pone.0142602 (PMC4641700; doi:10.1371/journal.pone.0142602)
Supplement: S2 Table — (DOCX) [file pone.0142602.s002.docx]

**S2 Table. Coverage of RAD-seq and microsatellites discovery per sample.**

| Individuals/Population | Sequences longer than or equal to 80 bp | No. of reads containing microsatellites | Consensus sequences (putative loci) |
| --- | --- | --- | --- |
| *P. machrisii 1/*DEL | 32,806 | 961 | 135 |
| *P. machrisii 2/*DEL | 27,396 | 658 | 267 |
| *P. machrisii 3/*DEL | 30,777 | 1,077 | 157 |
| *P. machrisii 4/*DEL | 31,060 | 969 | 131 |
| *P. machrisii 5/*DEL | 52,083 | 1,715 | 254 |
| *P. machrisii 6/*CRI | 11,312 | 358 | 39 |
| *P. machrisii 7/*CRI | 39,224 | 1,204 | 142 |
| *P. machrisii 8/*CRI | 32,642 | 1,200 | 168 |
| *P. machrisii 9/*CRI | 35,592 | 1,133 | 146 |
| *P. machrisii 10/*CRI | 41,153 | 1,533 | 200 |
| *P. machrisii 11*/ART | 32,659 | 1,819 | 191 |
| *P. machrisii 12/*ART | 37,026 | 1,221 | 174 |
| *P. machrisii 13/*ART | 31,021 | 1,107 | 135 |
| *P. machrisii 14/*ART | 29,308 | 1,144 | 112 |
| *P. machrisii 15/*ART | 24,479 | 827 | 88 |
| *P. machrisii 16/*APA2 | 31,249 | 1,173 | 136 |
| *P. machrisii 17/*APA2 | 43,451 | 1,568 | 178 |
| *P. machrisii 18/*APA2 | 35,063 | 1,493 | 155 |
| *P. machrisii 19/*APA2 | 32,755 | 1,351 | 124 |
| *P. machrisii 20/*APA2 | 36,497 | 1,181 | 176 |
| *P. aurisetus 1/*MEN | 64,041 | 2,150 | 384 |
| *P. aurisetus 2/*MEN | 42,176 | 1,700 | 152 |
| *P. aurisetus 3/*MEN | 40,459 | 1,764 | 252 |
| *P. aurisetus 4/*MEN | 40,497 | 1,310 | 177 |
| *P. aurisetus 5/*MEN | 37,427 | 1,571 | 209 |
| *P. aurisetus 6/*GMO | 28,957 | 1,398 | 154 |
| *P. aurisetus 7/*GMO | 25,974 | 803 | 101 |
| *P. aurisetus 8/*GMO | 24,368 | 503 | 64 |
| *P. aurisetus 9/*GMO | 42,158 | 1,170 | 137 |
| *P. aurisetus 10/*GMO | 42,300 | 1,352 | 181 |
| *P. vilaboensis 1/*PIR | 19,010 | 803 | 92 |
| *P. vilaboensis 2/*PIR | 33,119 | 1,692 | 156 |
| *P. vilaboensis 3/*PIR | 35,632 | 1,588 | 176 |
| *P. vilaboensis 4/*PIR | 44,763 | 1,891 | 169 |
| *P. vilaboensis 5/*PIR | 45,777 | 1,762 | 186 |
| *P. jauruensis 1/*RVE | 39,998 | 2,125 | 206 |
| *P. jauruensis 2/*RVE | 49,251 | 2,084 | 265 |
| *P. jauruensis 3/*RVE | 33,140 | 1,763 | 149 |
| *P. jauruensis 4/*RVE | 39,175 | 1,975 | 156 |
| *P. gounellei* | 45,136 | 1,324 | 135 |
| Total | 1,440,911 | 54,420 | 6,609 |

Populations code: DEL=Delfinópolis-MG, CRI=Cristalina-GO, ART=Aurora do Tocantins-TO, APA2=Alto Paraíso de Goiás-GO, MEN=Mendanha-MG, GMO=Grão Mogol-MG, PIR=Pirenópolis-GO, RVE=Rio Verde de Mato Grosso-MS.
